# Efficient recognition of dynamic user emotions based on deep neural networks

**DOI:** 10.3389/fnbot.2022.1006755

**Published:** 2022-09-29

**Authors:** Qi Zheng

**Affiliations:** School of Communication, Zhengzhou Normal University, Zhengzhou, China

**Keywords:** deep learning models, dynamic users, sentiment analysis, text extraction, tag semantics

## Abstract

The key issue at this stage is how to mine the large amount of valuable user sentiment information from the massive amount of web text and create a suitable dynamic user text sentiment analysis technique. Hence, this study offers a writing feature abstraction process based on ON-LSTM and attention mechanism to address the problem that syntactic information is ignored in emotional text feature extraction. The study found that the Att-ON-LSTM improved the micro-average F1 value by 2.27% and the macro-average F value by 1.7% compared to the Bi-LSTM model with the added attentivity mechanisms. It is demonstrated that it can perform better extraction of semantic information and hierarchical structure information in emotional text and obtain more comprehensive emotional text features. In addition, the ON-LSTM-LS, a sentiment analysis model based on ON-LSTM and tag semantics, is planned to address the problem that tag semantics is ignored in the process of text sentiment analysis. The experimental consequences exposed that the accuracy of the ON-LSTM and labeled semantic sentiment analysis model on the test set is improved by 0.78% with the addition of labeled word directions compared to the model Att-ON-LSTM without the addition of labeled semantic information. The macro-averaged F1 value improved by 1.04%, which indicates that the sentiment analysis process based on ON-LSTM and tag semantics can effectively perform the text sentiment analysis task and improve the sentiment classification effect to some extent. In conclusion, deep learning models for dynamic user sentiment analysis possess high application capabilities.

## Introduction

In recent years, online social media and mobile smart terminals have emerged in large numbers and developed rapidly. They provide people with new communication process and interactive spaces, making people's lifestyles change dramatically (Aprem and Krishnamurthy, 2017). A growing number of people tend to use smart terminals to obtain information, exchange ideas and spread information through online social media. The process of information dissemination is no longer the one-way communication of traditional media, but interactive communication (Nduhura and Prieler, 2017). Among them, there is a large amount of text as the simplest and most direct carrier for human beings to express their thoughts and spread knowledge in the Internet. It involves hot events, product reviews, news information and many other aspects. They contain rich emotional information and attitudinal views, with high social value and commercial application value (Liu et al., 2017). Emotion, a physiological and psychological state that results from a combination of feelings, thoughts, and behaviors (Chao et al., 2017). Such as happiness, sadness and anger are also among the chief factors that influence human behavior. People discuss certain hot topics and express their opinions on social platforms. They publish reviews of products or services on shopping platforms, etc. These unstructured online texts contain a lot of valuable information about users' emotions (Angioli et al., 2019). These emotional messages inherently have a certain abstraction and are difficult to be processed directly. It has attracted the attention of many researchers to find out how to extract this effective information from the huge amount of web texts and then apply it effectively in real life, resulting in the emergence of dynamic user text sentiment analysis techniques.

Initial studies on sentiment analysis focused on coarse-grained research on sentiment polarity dichotomization or trichotomization. With the gradual research on text sentiment analysis techniques and the desire to have a more comprehensive understanding of users' psychological states, the study of text sentiment analysis gradually shifted to more fine-grained multi-categorization research. Emotion recognition is the foundation and prerequisite of emotion classification. In the vast amount of realistic web texts, there are filled with abundant unemotional texts and emotional texts. Emotionless text is an objective description of things or events without any emotion. Emotional texts contain personal emotions such as happiness, anger, sadness and so on. Emotional texts are the main object of textual emotion analysis, therefore, it is necessary and chief to identify the presence or absence of emotions in a large amount of real texts and filter out emotional texts. Sachdev et al. (2020) collected a corpus of blog posts, which were annotated with word-level emotion categories and intensities, and used a knowledge-based approach to identify sentences with and without emotions with an accuracy of 73.89%. Wallgrün et al. (2017) constructed a corpus oriented to microblogging texts, annotated whether the microblogging texts contain emotion information or not. At the same time, they performed a multi-label annotation of the emotion categories contained in the microblog texts with emotions. They summarized the results of the NLP&CC2013 Chinese microblog sentiment analysis evaluation task on sentiment recognition, which facilitated the research related to sentiment analysis. Huang et al. (2017) planned a text emotion recognition process based on syntactic information, which expands the performance of emotion recognition by making full use of syntactic information through lexical annotation sequences and syntactic trees. Emotion classification, as chief research direction of emotion analysis, is based on emotion recognition to classify texts containing emotion information at a finer granularity and obtain the specific emotion category (e.g., happy, angry, sad, etc.) expressed by the user in the text. The majority of early sentiment classification studies utilized lexicon- and rule-based process to determine sentiment categories. Fine-grained multi-class sentiment classification is the difficulty and focus of sentiment analysis, researchers have studied sentiment classification from different perspectives, such as construction of sentiment corpus (Fraser and Liu, 2014; Kawaf and Tagg, 2017), author sentiment and reader sentiment prediction (Chang et al., 2015; Yoo et al., 2018), document-level sentimentality organization, sentence-level sentimentality organization and word-level sentiment classification (Liu et al., 2020; Zhang, L., et al., 2021). Alves and Pedrosa (2018) planned a process based on frequency and co-occurrence information to classify the sentiment of headline texts by making full use of the co-occurrence relationship between contextual words and sentiment keywords. Subsequently, various researchers have used traditional machine learning-based process for sentiment classification studies. Pan et al. (2017) planned a multi-label K-nearest neighbor (KNN) based sentiment classification process that explores the polarity of words, sentence subject-verb-object components, and semantic frames as features for their impact on sentiment classification. To differ from this, Aguado et al. (2019) have taken into accounts the interaction of sentiments between sentences and use a coarse-to-fine analysis strategy to do sentiment classification of sentences, first obtaining the set of possible sentiments embedded in the target sentence roughly using a multi-label K-nearest neighbor approach, and then refining the sentiment category of the target sentence by combining the sentiment transfer probabilities of neighboring sentences. Rao et al. (2016) utilized a maximum entropy model to model words and multiple sentiment categories in a text to estimate the relationship between words and sentiment categories to classify sentiment in short texts. Traditional machine learning process extract text features manually through feature engineering, while deep learning uses representation learning process to extract text features automatically without relying on artificial features. Effective feature extraction is the core of research on emotion classification process, and most of research works show that reasonable use of deep learning techniques based on neural networks to extract rich semantic information in emotional texts contributes to the effectiveness of text emotion classification. Abdul-Mageed and Ungar (2017) constructed a large-scale fine-grained sentiment analysis dataset employing Twitter data and designed a represent neural network based on gating units to achieve 24 classes of fine-grained sentiment classification. Kim and Huynh (2017) experimentally explored text emotion classification utilizing the LSTM model as well as its variant nested long-short-term memory network (Nested LSTM) model, respectively, which showed that the Nested LSTM model facilitates better accuracy of sentiment classification. Wang et al. (2016) planned neural network (NN) model based on bilingual attentively mechanisms for the problem of emotion classification in bilingual mixed text, where the LSTM model is used to construct a document-level text illustration and the attention mechanism captures semantically rich words in both monolingual and bilingual texts. In addition, some studies have applied a joint multi-task learning approach to the task of emotion classification. Awal et al. (2021) incorporated emotion classification and emotion cause detection as two subtasks into a unified framework through a joint learning model, trained simultaneously to extract the emotional features needed for emotion classification and the event features needed for emotion cause detection. Yu et al. (2018) planned a dual-attention-based transfer learning process that aims to improve the performance of emotion classification using sentiment classification. At present, there are plenty of results for research on text sentiment analysis process, but sentiment analysis still faces many challenges due to the colloquial and irregular nature of online texts and the complexity of sentiment itself (Ning et al., 2021). For text representation, most approaches use pre-trained models such as Word2Vec and GloVe to obtain word directions, which are simple, efficient, and can characterize contextual semantics well, but suffer from the problem of multiple meanings of a word (Ning et al., 2022). For text feature extraction, commonly used NNs such as CNN and Bi-LSTM extract semantic features while ignoring the syntactic hierarchy features of the text (Ning et al., 2020). Most approaches only symbolize sentiment category labels and act as a supervisory role in the classification process, while ignoring semantic information contained in the labels themselves, which is undoubtedly a “semantic waste”. In this paper, we explore and improve the text sentiment analysis process based on the above three problems.

Based on this, the main research of this paper is to use deep learning techniques to accomplish the task of emotional analysis of online text, based on the ordered neuronal long and short term memory network (ON-LSTM) and attention mechanism, and incorporating the semantic information of emotional category labels to build the emotional analysis model ON- LSTM-LS. First, the text features are extracted based on ON-LSTM and attention mechanism. Then the sentiment analysis model based on ON-LSTM and label semantics. In this study, for online social text, we build the sentiment analysis model ON-LSTM-LS based on ordered neuron long and short term memory network and attention mechanism, and incorporate the semantic information of sentiment category labels to improve the performance of text sentiment analysis.

## Theory and model construction

### Text representation

Text data usually consists of a set of unstructured or semi-structured strings. Since computers cannot directly recognize and process text strings, they need to numerate or directionize the text, i.e., text representation. Text representation enables computers to process real text efficiently and is a fundamental and chief step in the study of text sentiment analysis. In Chinese, words are generally considered to be the most basic semantic units of text. Therefore, general research for Chinese text should first perform word separation operation, and then the words in the text are represented afterwards.

#### Word direction representation

NN-based distributed representation is also recognized as word direction, word embedding or distributed representation of words. This process models the target word, context of target word and the relationship between them, and represents the target word as a low-dimensional solid real-valued direction in continuous space. Compared with matrix-based distributed representation and cluster-based distributed representation, word direction representation can contain more and more complex semantic information. It is extensively used in various normal language dispensation errands.

The word directions are gained by training the language model, which uses a single-layer NN to perform the solution of the binary language model while obtaining the word direction representation. Based on this, the NN language model NNLM is planned (Wang et al., 2022). The model takes the first *k* words of the present word *w*_*t*_, *w*_*t*−*k*−1_, …, *w*_*t*−1_, as input and uses a NN to predict the conditional likelihood of the occurrence of the present word *w*_*t*_ to obtain a word direction representation while training the language model. As the NNLM is capable of handling only fixed-length sequences, lacking flexibility. Due to the slow training speed, the researchers improved the NNLM. They planned two words direction training models, which are successive bag-of-words model CBOW and skip-word model. They open source a tool Word2Vec for word direction computation. Differs from the NNLM in which the representation of the present word *w*_*t*_ depends on its predecessor. In CBOW and Skip-gram models, the representation of the present word *w*_*t*_ depends on *k* words before and after it. In the CBOW and Skip-gram models, representation of present word *w*_*t*_ depends on *k* words before and after it.

**Figure 1:**
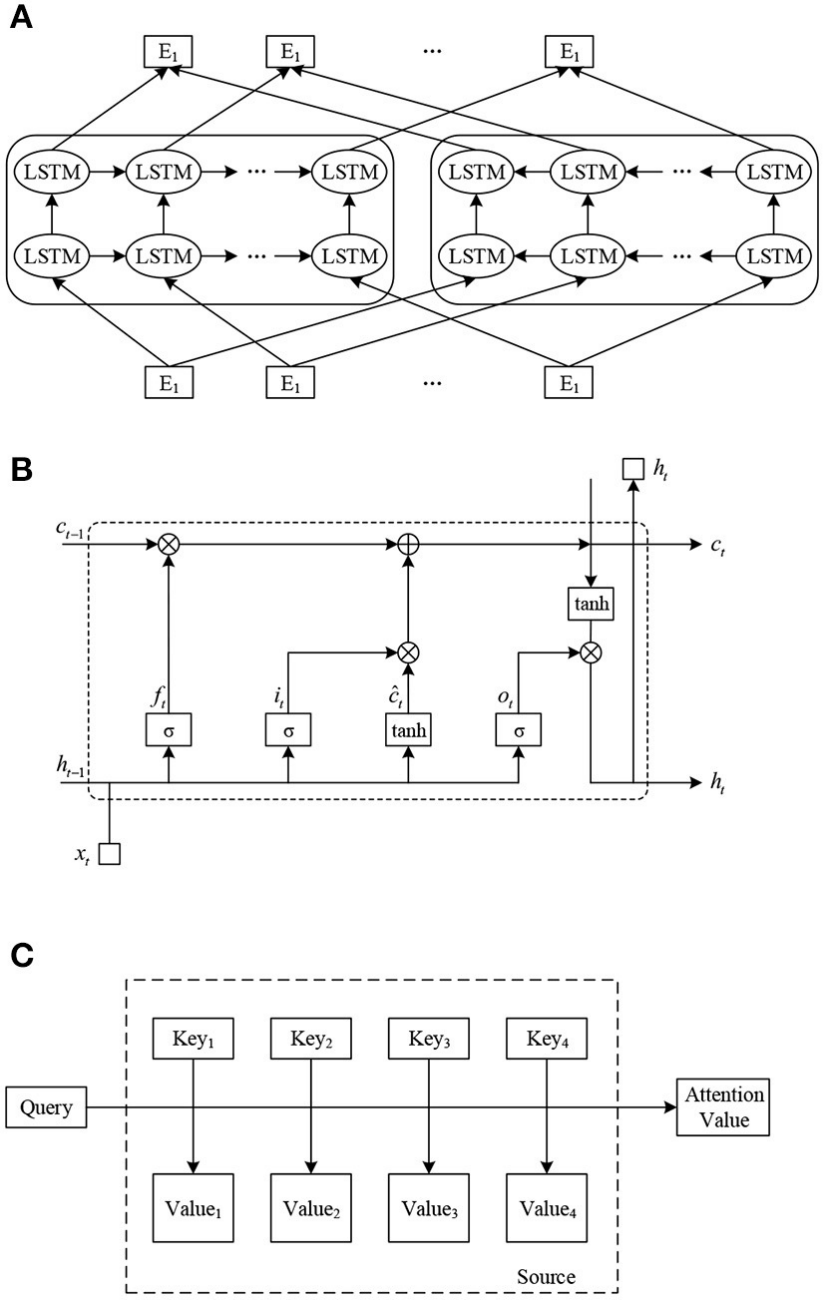
Model structure diagram. **(A)** ELMo model structure diagram. **(B)** LSTM model structure diagram. **(C)** Attention model structure diagram.

Both CBOW and Skip-gram models have same three-layer hierarchy: input layer, mapping layer, output layer, and structure diagram are exposed in Figure 1A. In the input layer, the input words are randomly initialized as N-dimensional directions. They enter the hidden layer after a simple linear operation, and then likelihood distribution of the target words is output by hierarchical Softmax.

The CBOW model is to predict the provisional likelihood of the occurrence of the word *w*_*t*_ by the context {*w*_*t*−*k*_, ⋯, *w*_*t*−1_, *w*_*t*+1_, ⋯, *w*_*t*+*k*_} of present word *w*_*t*_, which is calculated as:


(1) $$\begin{array}{l} p\left(w_{t}|c_{t}\right)=\frac{\exp\left(e\prime {\left(w_{t}\right)}^{T}x\right)}{\overset{\left|V\right|}{\underset{i=1}{\sum }}\exp\left(e\prime {\left(w_{i}\right)}^{T}x\right)} \end{array}$$

(2) $$\begin{array}{l} x=\underset{i\in k}{\sum }e\left(w_{i}\right) \end{array}$$

where *c*_*t*_ denotes the words {*w*_*t*−*k*_, ⋯, *w*_*t*−1_, *w*_*t*+1_, ⋯, *w*_*t*+*k*_} in the context window, *e*(*w*_*t*_) is input direction of word *w*_*t*_, *e′*(*w*_*t*_) is output direction of word *w*_*t*_, *x* is the context direction, *V* is the corpus word list.

The Skip-gram model uses the present word *w*_*t*_ to predict the conditional likelihood of each word in the vocabulary to occur in its context (Wang et al., 2021) and is calculated as:


(3) $$\begin{array}{l} p\left(w_{j}|w_{t}\right)=\frac{\exp\left(e\prime {\left(w_{j}\right)}^{T}e\left(w_{t}\right)\right)}{\sum_{i=1}^{\left|V\right|}\exp\left(e\prime {\left(w_{i}\right)}^{T}e\left(w_{t}\right)\right)} \end{array}$$

To limit the number of restrictions to restore the training efficiency of the model, Word2Vec is optimized using two techniques, hierarchical Softmax and negative sampling. In the training process of model, representation of words is constantly updated by the output direction of the mapping layer is the direction of words.

Considering that Word2Vec only considers contextual co-occurrence features within a finite window when learning, global statistical features are ignored. Researchers propose the GloVe word embedding technique that fuses global and local contextual features of text. gloVe belongs to matrix-based distribution representation, which is a global logarithmic bilinear regression model. They construct global word-word co-occurrence matrices based on the statistical information between words in the corpus. They use a global matrix decomposition and local context window approach for unsupervised training of non-zero positions in the co-occurrence matrix, and a back-propagation algorithm to solve the word directions. Compared with Word2Vec, GloVe has stronger scalability.

Word2Vec and GloVe use pre-training techniques to represent each word as a word direction with a contextual semantic representation. They solve the problems of sparse data, dimensional disaster, and lack of semantic information representation that exist in traditional one-hot directions. At the same time, they have become the mainstream text representation techniques because of their simplicity and efficiency. They have greatly contributed to the development of natural language processing research.

#### Dynamic word direction technique

While the word direction representation techniques, represented by Word2Vec and GloVe, have greatly advanced the development of natural language processing, they also have some problems. One of the biggest problems is polysemy. Word2Vec and GloVe learn word directions as static word directions, i.e., the word-direction relationship is one-to-one. In other words, no matter how the context of a word changes, the trained word direction is uniquely determined and does not change in any way as the context changes. This static word direction cannot solve the problem of multiple meanings of a word. To solve the problem, researchers have conducted some exploratory research. They have planned a dynamic word direction technique based on language model. Dynamic word directions are not fixed, but change at any time according to the contextual background. ELMo, a language model that can be used to train dynamic word directions, is presented next.

ELMo (Embedding from Language Models) is a novel deep contextualized word representation model planned by Peters et al. By training the model, high quality dynamic word directions can be gained. The model is trained using a deep bi-directional language model (biLM) to obtain different word representations for different contextual inputs, i.e., to generate word directions dynamically.

ELMo uses a two-stage training process. The first stage is to pre-train a language model on a large corpus using a multilayer biLM before training on a specific task. This language model is equivalent to a “dynamic word direction generator” that generates specific word directions for a specific task. In the second stage, the word directions generated by the language model in the first stage are added to the downstream task as a feature supplement for task-specific training. The model structure of ELMo is exposed in Figure 1B. Where (E_1_, E_2_, …, E_N_) are the static Word Embedding of the input word sequence, (T_1_, T_2_, …, T_N_) are the dynamic Word Embedding of the output gained by pre-training the ELMo model.

The ELMo model employs bivectorial LSTM language model to train word representation in the first stage. Specifically, suppose that given a word sequence (*w*_1_, *w*_2_, …, *w*_*N*_) of length *N*, the forward LSTM language model calculates the sequence likelihood of the occurrence of the word at the 1, 2, …, *k*−1 by taking the word sequence at the given first position as:


(4) $$\begin{array}{l} p\left(w_{1},w_{2},\ldots ,w_{N}\right)=\overset{N}{\underset{k=1}{\prod }}p\left(w_{k}|w_{1},w_{2},\ldots ,w_{k-1}\right) \end{array}$$

The backward LSTM language model calculates the sequence likelihood of the occurrence of words at position *k* + 1, *k* + 2, …, *k* + *N* by taking the sequence of words at position *k* given as:


(5) $$\begin{array}{l} p\left(w_{1},w_{2},\ldots ,w_{N}\right)=\overset{N}{\underset{k=1}{\prod }}p\left(w_{k}|w_{k+1},w_{k+2},\ldots ,w_{N}\right) \end{array}$$

The combination of frontward LSTM language model and backward LSTM language model constitutes the bi-directional language model biLM, which is required to maximize the following objective function during the training process as:


(6) $$\begin{array}{l} \overset{N}{\underset{k=1}{\sum }}\left(\begin{array}{c} \log p\left(w_{k}|w_{1},w_{2},\ldots ,w_{k-1};\Theta_{x},{\vec{\Theta }}_{lstm},\Theta_{s}\right)\\ +\log p\left(w_{k}|w_{k+1},w_{k+2},\ldots ,w_{N};\Theta_{x},{\overset{\leftarrow }{\Theta }}_{lstm},\Theta_{s}\right) \end{array}\right) \end{array}$$

where Θ_*x*_ denotes the word direction matrix, Θ_*x*_ is the parameter of the softmax layer, ${\vec{\Theta }}_{lstm}$ and ${\bar{\Theta }}_{lstm}$ denote frontward LSTM language model and the backward LSTM language model, respectively.

The L-layer biLM model is used in the pre-training, and the word representations gained from each layer have different features. For each input word, the word representation with features such as syntactic semantics is output for it by pre-training with the L-layer biLM model as:


(7) $$\begin{array}{l} R_{k}=\left\{x_{k}^{LM},{\vec{h}}_{k,j}^{LM},{\bar{h}}_{k,j}^{LM}|j=1,\ldots ,L\right\}\\ \;=\left\{h_{k,j}^{LM}|j=0,\ldots ,L\right\} \end{array}$$

where $h_{k,0}^{LM}$ is the word layer, $h_{k,j}^{LM}=\left|{\vec{h}}_{k,j}^{LM},{\overset{\leftarrow }{h}}_{k,j}^{LM}\right|$.

A language model and word representations for each hidden layer will be gained after the first stage of training. In the second stage, the sentences in the downstream task will be used as input to the dimensional ELMo. For each word in the sentence, ELMo combines the word representations of all hidden layers into a direction by calculating the weights of each hidden layer. That is, a direction of words in the present context. It is formalized as:


(8) $$\begin{array}{l} {\mathrm{ELMo}}_{k}^{\text{task}}=E\left(R_{k};\Theta^{\text{task}}\right)=\gamma^{\text{task}}\overset{L}{\underset{j=0}{\sum }}s_{j}^{\text{task}}h_{k,j}^{LM} \end{array}$$

where γ^task^ is the validated global scaling factor and $s_{j}^{\text{task}}$ is the softmax normalized weighting factor.

ELMo is able to dynamically generate different word directions for the similar word in dissimilar circumstances. It conforms to the word direction of the present context. It solves the problem of encoding multiple meanings of a word to some extent and performs well in several natural language processing tasks.

### Deep learning models

The concept of deep learning (DL) (Wang et al., 2021) originated from the study of artificial NNs. It involves of multiple layers of artificial NNs connected to be able to extract effective feature representation information from a large amount of input data. Deep learning mimics the way the human brain operates. It learns from experience and has the ability to excel in representation learning. It has been successfully applied in several research fields.

#### Represent NNs

Represent neural network (RNN) is a NN with short-term memory capability to process temporal information of varying lengths. It is widely used for several tasks in natural language processing. RNNs use neurons with self-feedback to process temporal information of arbitrary length by unfolding multiple times. Where *x*_*t*_ denotes input direction at the time of *t*, *h*_*t*_ denotes state of the hidden layer at the time of *t*. *o*_*t*_ denotes output direction at the time of *t*. *U* denotes Input layer to hidden layer weight matrix, *V* denotes Value matrix of weights from hidden layer to output layer. *W* denotes value of the weight matrix of the preceding moment of hidden layer as input value of the current moment.

The RNN gives an output *o*_*t*_ with present network hidden layer state *h*_*t*_ for input *x*_*t*_ at *t*. The value of *h*_*t*_ at time *t* depends upon not only *x*_*t*_, as well as also on the hidden layer state *h*_*t*−1_ at previous moment, calculated as:


(9) $$\begin{array}{l} o_{t}=g\left(Vh_{t}\right) \end{array}$$

(10) $$\begin{array}{l} h_{t}=f\left(Ux_{t}+Wh_{t-1}\right) \end{array}$$

where *f* represents a non-Linear activation function such as sigmod or tanh. The network parameters are shared at different moments and are trained by the backpropagation over time algorithm (BPTT).

Theoretically, RNNs are capable of handling text sequences of arbitrary length. However, in practice, when the length of text sequences is too long, the problem of gradient explosion or gradient dispersion occurs. It makes parameter updating difficult, which in turn prevents RNNs from learning long-range dependency information. It also leads to biased learning results of long-range dependencies, i.e., RNNs learn short-term dependence.

#### Short long-term memory network

A variation of RNN, Long-Short-Term Memory Network (LSTM) can effectively tackle the problem of gradient explosion or gradient dispersion in RNN (Yan et al., 2021). The improvement of LSTM for RNN is twofold. On the one hand, during the training process of the network, RNN has only one state *h*_*t*_ at the moment *t*, LSTM adds a state *c*_*t*_ on this basis, *c*_*t*_ represents the memory state of the represent unit, which involves a small number of function operations and thus can store long distance information. On the other hand, LSTM introduces a gating mechanism and designs three gate structures, forgetting gate, input gate, and output gate, which enable represent NN to selectively forget some unchief information while remembering the past information through the interaction between the three gates, and thus learn longer distance dependencies.

The cell of LSTM has certain memory function because of it. It also called a memory cell. The structure of the LSTM loop cell is exposed in Figure 1B. where *x*_*t*_ represents input direction of the memory cell at moment *t*. *h*_*t*_ represents output direction of the memory cell at time *t*. represents present information after updating the memory. *f*_*t*_, *o*_*t*_ and *i*_*t*_ represent the forgetting gate, output gate and input gate, respectively.

The forgetting gate *f*_*t*_ selectively forgets a portion of the cell state information through the sigmod layer, i.e., it determines how much of the cell condition *c*_*t*−1_ of previous moment needs to be retained in the cell state *c*_*t*_ of the present moment. The calculation formula is as follows:


(11) $$\begin{array}{l} f_{t}=\sigma \left(W_{f}x_{t}+U_{f}h_{t-1}+b_{f}\right) \end{array}$$

Input gate *i*_*t*_ selectively records new inputs in the memory cells through the sigmod layer. It determines how much of the present network's input *x*_*t*_ needs to be saved into the cell state *c*_*t*_. Also, the cell state *c*_*t*_ of the present input is determined based on the output *h*_*t*−1_ of hidden layer state and *x*_*t*_ at the present moment, which is calculated as follows:


(12) $$\begin{array}{l} i_{t}=\sigma \left(W_{i}x_{t}+U_{i}h_{t-1}+b_{i}\right) \end{array}$$

(13) $$\begin{array}{l} {ĉ}_{t}=tanh\left(W_{c}x_{t}+U_{c}h_{t-1}+b_{c}\right) \end{array}$$

The present memory ĉ_*t*_ and the historical memory *c*_*t*−1_ need to be combined before the output gate to update the cell state *c*_*t*_ at the present moment. The forgetting gate allows chief information from long ago to be preserved, and the input gate allows irrelevant information from the present input to be filtered and forgotten.


(14) $$\begin{array}{l} f_{t}=\sigma \left(W_{f}x_{t}+U_{f}h_{t-1}+b_{f}\right) \end{array}$$

The output gate *o*_*t*_ inputs the input information and the present cell state update to the next hidden layer *h*_*t*_ through the sigmod layer, i.e., it controls how much of the cell state *c*_*t*_ needs to be output to the hidden layer state *h*_*t*_ at the present moment. The calculation formula is as follows:


(15) $$\begin{array}{l} o_{t}=\sigma \left(W_{o}x_{t}+U_{o}h_{t-1}+b_{o}\right) \end{array}$$

(16) $$\begin{array}{l} h_{t}=o_{t}◦tanh\left(c_{t}\right) \end{array}$$

In the above equations, *W*_*f*_, *W*_*i*_ and *W*◦ are weight matrices from input layer to the forgetting gate, input gate and output gate, respectively. *U*_*f*_, *U*_*i*_ and *U*_*o*_ are weight matrices from output layer to the forgetting gate, input gate and output gate, respectively. *W*_*c*_ is connection weight from input layer to the LSTM loop unit. *U*_*c*_ is the connection weight from the previous node to the present node of the LSTM loop unit, *b*_*f*_, *b*_*i*_, *b*_*o*_ and *b*_*c*_ are all offsets.

Weight parameters in RNN are shared across time steps, which is why there is gradient explosion or dispersion. In contrast, there are multiple paths of gradient propagation in LSTM. In which the process of cell state update at the present moment is carried out by element-by-element multiplication and summation. Its gradient flow is relatively stable, thus greatly reducing the risk of gradient explosion or dispersion. Thus, LSTM is able to handle long-range temporal information.

The input gate determines degree of retention of the input information. The forgetting gate determines the extent to which memory information is forgotten. The output gate, on the other hand, controls the extent to which internal memory is output to the outside. Each of the three gating switches has its own role, which enables LSTM to effectively use historical information and establish long-range temporal dependencies. In turn, it is widely used in tasks related to sequential problems.

### Gated circulation unit

Gated represent unit (GRU) is a represent NN based on another gating mechanism. The basic design idea of GRU is the same as LSTM, and it can be said to be a variant of LSTM. Its difference lies in two main aspects. On the one hand, the structure of GRU is relatively simple, using two gate structures. The reset gate determines how much historical information requires to be forgotten. The update gate determines how much of the history information can be saved to the present state. On the other hand, GRU directly passes the hidden state to the next cyclic unit without using an output gate.

Where *x*_*t*_ represents input direction at the present moment, *h*_*t*−1_ represents state at previous moment, ${\tilde{h}}_{t}$ is the candidate state at the present moment, *r*_*t*_ and *z*_*t*_ represent reset gate and update gate, respectively, and output direction *h*_*t*_ at the present moment is calculated as in Eqs. (17–20).


(17) $$\begin{array}{l} r_{t}=\sigma \left(W_{r}x_{t}+U_{r}h_{t-1}\right) \end{array}$$

(18) $$\begin{array}{l} z_{t}=\sigma \left(W_{z}x_{t}+U_{z}h_{t-1}\right) \end{array}$$

(19) $$\begin{array}{l} {\tilde{h}}_{t}=tanh\left(W_{h}x_{t}+U_{h}\left(r_{t}◦h_{t-1}\right)\right) \end{array}$$

(20) $$\begin{array}{l} h_{t}=\left(1-z_{t}\right)◦h_{t-1}+z_{t}◦{\tilde{h}}_{t} \end{array}$$

Reset gates help capture short-term dependencies. The update gate, on the other hand, helps to capture long-term dependencies. Therefore, GRU can handle long and short-term dependencies in sequences. At the same time, GRU has one less gate structure compared to LSTM, and the number of parameters is relatively less, and the overall training speed is improved.

### Attentional mechanisms

The Attention mechanism assigns higher weights to task-relevant information by weighting (Zhang, M., et al., 2021). While assigning lower weights to task-irrelevant information, and then filtering the relatively chief information from the large amount of information. Introducing the attention mechanism into machine translation tasks in the natural language domain achieves significant effect improvement. Since then, the attention mechanism has received some attention in natural language processing. Researchers have combined it with DNN to extract features that are more relevant to the task and thus expand the performance of the model. For example, in sentiment classification, a set of directions or matrices with parameters are used to characterize the importance of words in a text sentence. In the process of extracting features, the features that are more relevant to the sentiment classification are extracted based on the importance of the words.

The core idea of the attention mechanism is to move from “focus on all” to “focus”. It focuses the limited attention on the chief information related to the task, so that effective information can be gained quickly. As exposed in Figure 1C, the Source can be observed as the content deposited in a memory, whose rudiments consist of (address Key, value Value), and given a query with Key=Query, the Value conforming to the query is removed, i.e., the Attention value. The specific calculation process is:

(1) The similarity between the Query and each Key is calculated as the weight coefficient of the Value conforming to each Key. The similarity is usually calculated by dot product, cosine, multilayer perceptron network, etc.
  (21) $$\begin{array}{l} Sim_{i}=F\left(Query,\;Key_{i}\right) \end{array}$$
(2) The similarity gained in the previous stage is normalized using softmax and transformed into a similarity whose sum of all similarity weights is 1, thus highlighting the weights of chief elements. The calculation formula is as follows, and is the weight coefficient conforming to each value.
  (22) $$\begin{array}{l} \alpha_{i}=softmax\left(Sim_{i}\right)=\frac{e^{Sim_{i}}}{\sum_{j=1}^{L_{x}}e^{Sim_{j}}} \end{array}$$
(3) The normalized weighting coefficients are weighted and summed with the conforming Value to obtain the final attention value.
  (23) $$\begin{array}{l} \;Attention\;\left(Query,\;Source\right)=\sum_{i=1}^{L_{x}}\alpha_{i}\cdot Value_{i} \end{array}$$
  Presently, the values of Key and Value are same in the research for natural language processing. And in the commonly used self-attention mechanism (self-Attention), Query (Q), Key (K) and Value (V) are all from the same input and all three are the same, noted as Q = K = V. In text analysis, for example, a sentence is input, and the self-attention mechanism requires that each word in the sentence is computed with all the words in the judgment to learn the dependences among the words within the sentence. The computation is formalized as:
  (24) $$\begin{array}{l} Attention\left(Q,K,V\right)=softmax\left(\frac{QK^{T}}{\sqrt{d_{k}}}\right)V \end{array}$$
  where *d*_*k*_ is the dimension of Q and K directions, $\sqrt{d_{k}}$ is a scaling factor, and the same division of $\sqrt{d_{k}}$ is to avoid the softmax gradient from decreasing or even disappearing due to excessive dot product.

## Experiment and analysis

### ON-LSTM-A based text feature extraction process

To confirm the dependability of the experimental consequences, this chapter uses the public dataset provided by the NLP&CC2013 Chinese microblog sentiment analysis evaluation task. The dataset is derived from Sina Weibo, with a total of 14,000 microblog texts. The dataset is divided into 7 categories of emotions and labeled with “none” for the text without emotions. In this chapter, the experiment classifies the text with emotion, so the text without emotion is removed. The distribution of the number of each type of emotional samples in the experimental data set is exposed in Table 1. From them, 60% were arbitrarily designated as the training set and 40% as the test set for the experiments.

**Table 1:** Emotional sample data statistics table.

**Emotional category**	**Happiness**	**Sadness**	**Joy**	**Disgust**	**Anger**	**Fear**	**Surprise**
Number of samples	1,487	1,132	2,155	1,360	671	151	348

This experiment uses the same evaluation metrics as the evaluation task: macro-averaged F1 values (*Macro*_*F measure*_) and micro-averaged F1 values (*Macro*_*F measure*_), which are calculated as follows:


(25) $$\begin{array}{l} Macro_{Percision}=\frac{1}{7}\underset{i}{\sum }\frac{\;\#\text{system\_orrect}\left(emotion=i\right)}{\;\#\text{system\_roposed}\left(emotion=i\right)} \end{array}$$

(26)  MacroRecall =17∑i #system_orrect(emotion=i)#gold(emotion=i)

(27) $$\begin{array}{l} \;Macro_{F_{-}measure\;}=\frac{2\times Macro_{Percision}\times Macro_{Recall}}{\;Macro_{Percision}+Macro_{Recall}} \end{array}$$

(28) $$\begin{array}{l} \;Micro_{Percision}=\frac{\sum_{i}\#\text{system\_orrect}\left(emotion=i\right)}{\sum_{i}\#\text{system\_roposed}\left(emotion=i\right)} \end{array}$$

(29) $$\begin{array}{l} \;Micro_{Recall}=\frac{\sum_{i}\#\text{system\_correct}\left(emotion=i\right)}{\sum_{i}\#gold\left(emotion=i\right)} \end{array}$$

(30) $$\begin{array}{l} Micro_{F\text{\_}measure}=\frac{2\times Micro_{Percision}\times Micro_{Recall}}{Micro_{Percision}+Micro_{Recall}} \end{array}$$

Where #gold is the number of manually labeled results, #system_correct is the number of correctly classified tweets, #system_planned is the total number of tweets forecasted by the model for the present category, *i* is one of the seven categories of sentiment.

To confirm the effectiveness of the Att-ON-LSTM model planned in this study, a comparison experiment with the following similar models was designed.

LSTMl, which encodes text sequences from front to back in a unidirectional manner. Only the influence of the above information on the below information is considered, and the contextual semantic features of the text sequences are extracted.

Bi-LSTM, encoding text sequences from both positive and negative directions, extracting to the relationship before and after the text, for the more reasonable text sequence features.

ON-LSTM: ordered neuron long and short-term memory network, capable of extracting text semantics along with hierarchical syntactic information of text to obtain comprehensive text features.

Bi-LSTM+Attention, which adds attention mechanism to Bi-LSTM to extract contextual semantic features, making the model focus more on the features of words related to classification.

ON-LSTM+Attention: that is, the sentiment analysis model Att-ON-LSTM based on ON-LSTM and attention mechanism planned in this chapter. Three layers of ON-LSTM network are used to extract text features, and attention mechanism is used to increase the attention of words related to classification. The results of the comparison experiments are exposed in Table 2.

**Table 2:** Comparison results of similar models.

**Models**	***Micro*_*F*_−_ *measure*_**	***Macro*_*F*_−_ *measure*_**
LSTM	0.3039	0.0665
Bi-LSTM	0.3008	0.2632
ON-LSTM	**0.3144**	**0.2781**
Bi-LSTM+Attention	0.3090	0.2893
ON-LSTM+Attention(ours)	**0.3317**	**0.2910**

The experimental results show that ON-LSTM has improved micro F1 values and macro F1 values compared to LSTM and Bi-LSTM. The ON-LSTM+Attention model, i.e., Att-ON-LSTM planned in this section, improves the micro-average F1 value by 2.27% and the macro-average F value by 1.7% compared with the Bi-LSTM model with the added attention mechanism. It is indicated that the text features extracted by Att-ON-LSTM are more comprehensive and can effectively improve the effect of text emotion classification. In this section, the Att-ON-LSTM model is experimentally validated by comparing it with similar baseline models. The study illustrates that the ON-LSTM network can better extract text features, which helps to improve the effect of sentiment analysis.

### Research on sentiment analysis process based on ON-LSTM and label semantics

#### Experimental environment and parameter setting

This chapter uses Google's open source deep learning framework TensorFlow to complete the experiment, the specific experimental environment configuration is shown in Table 3.

**Table 3:** Experimental environment configuration.

**Experimental environment**	**Configuration**
CPU	19
Memory	64GB
Video card	NVIDIA GTX 2080ti
Development languages	python 3.7.4
Deep learning tools	TensorFlow 1.15.0

The model parameter settings in the experiments of this chapter are adjusted according to the performance of the validation set, and the relevant parameter settings are shown in Table 4.

**Table 4:** Model-related parameter settings.

**Parameter name**	**Value**
Word vector dimension	768
Maximum number of words in a sentence	100
ON-LSTM training dimension	[100,128]
Learning rate	0.01
Dropout ratio	0.25
Batch	256
Number of iterations	30

#### Experimental data set

To ensure the reliability of the experimental results, the CLUE Emotion Analysis Dataset provided by the Chinese Language Understanding Benchmark Assessment was used for the experimental data in this chapter. The corpus in this dataset comes from Sina Weibo and contains a total of 39,661 emotion samples, each with an emotion category label. There are seven types of emotion category labels: happiness, sadness, like, disgust, anger, fear, and surprise. In this experiment, 80% of the dataset is selected. Serving as the training set, 10% being used as the validation set, while the remaining 10% being used as the test set, specific distribution of the sample data is shown in Table 5.

**Table 5:** Statistics on the distribution of samples in the data set.

**Emotional**	**Training**	**Validation**	**Test**	**Total**	**Percentage (%)**
**category**	**set**	**set**	**set**	
Happy	7,975	1,006	978	9,959	25.11
Sad	11,210	1,394	1,448	14,052	35.43
Pleasant	3,657	430	453	4,540	11.45
Disgusted	3,896	509	471	4,876	12.29
Anger	3,657	458	447	4,562	11.50
Fear	525	69	67	661	1.67
Surprise	808	100	103	1,011	2.55
Total	31,728	3,966	3,967	39,661	100.00

#### Evaluation indicators

The sentiment classification problem is a multi-category problem and the following metrics are used in this experiment: Accuracy, Precision P, Recall R and F1 value. Accuracy usually measures the performance of the model on the whole data set. Accuracy cannot be reflected when the model is biased and is always wrong in certain categories of judgments. Therefore, it needs to be judged for each category using the P, R, and F1 values, and then averaged, and thus judged for the model as a whole. There are two types of averaging: Macro-average and Micro-average. Macro-average considers each category equally, increasing the impact of categories with less data. Micro-average considers each sample to be classified equally and is more influenced by common categories. In order to better measure the classification effect of the model for each category, the F1 value of macro-average is chosen as the evaluation index in this paper.

For the entire data set, Accuracy is calculated as:


(31) $$\begin{array}{l} \;Accuracy=\frac{\sum_{i=1}^{7}a_{E_{i}}}{N} \end{array}$$

where $\sum_{i=1}^{7}a_{E_{i}}$ denotes the sum of the number of correctly forecasted samples in each category and N is the total number of samples in the dataset.

For the sentiment category, Precision, Recall and F1-measure are calculated as:


(32) $$\begin{array}{l} P=\frac{a}{a+c} \end{array}$$

(33) $$\begin{array}{l} R=\frac{a}{a+b} \end{array}$$

(34) $$\begin{array}{l} F1=\frac{2\cdot P\cdot R}{P+R} \end{array}$$

Macro-averaging treats each category equally and calculates the arithmetic mean of the indicators for each category as:


(35) $$\begin{array}{l} Macro-P=\frac{1}{7}\overset{7}{\underset{i=1}{\sum }}P_{i} \end{array}$$

(36) $$\begin{array}{l} Macro-R=\frac{1}{7}\overset{7}{\underset{i=1}{\sum }}R_{i} \end{array}$$

(37) $$\begin{array}{l} Macro-F1=\frac{2\times Macro-P\times Macro-R}{Macro-P+Macro-R} \end{array}$$

#### Analysis of experimental results

To confirm the effectiveness of the sentiment analysis model based on ON-LSTM and label semantics and its performance, two sets of experiments were designed in this study. The first group is an ablation experiment to confirm the effect of label semantics on the effect of sentiment classification. The second group is a similar model comparison experiment, which compares the ON-LSTM-LS model with other sentiment analysis models based on deep learning to confirm the effectiveness and performance of the ON-LSTM-LS model planned in this chapter.

A. Ablation experiment results and analysis.
  The set of experiments is based on the Att-ON-LSTM model in Chapter 3, and the semantics of labeled word directions, the semantics of labeled semantics expanded text, and optimization with a weighted loss function are added in turn to confirm the effect of labeled semantics on the effect of emotion classification. The contrasting models are as follows.
  (a) ON-LSTM + Attention: The model is Att-ON-LSTM, a sentiment analysis model based on ON-LSTM and attention mechanism in Section ON-LSTM-A based text feature extraction process it uses the combination of three-layer ON-LSTM network and attention mechanism to extract the sentiment features in the text as the baseline model for this group of experiments;
  (b) ON-LSTM + Attention + Label: add word direction semantic features of labeled words to the Att-ON-LSTM model. It is combined with the sentiment features of the text to guide the model for sentiment classification using label semantics;
  (c) ON-LSTM + Attention + Label + Extra: This model expands the label semantics using the label semantic expansion process. It obtains richer label semantic features and expects to improve the effect of emotion classification;
  (d) ON-LSTM + Attention + Label + Extra + Customloss: This model is the ON-LSTM-LS model planned in this chapter. The cross-entropy loss function with weights is used on the basis of the model in (3) to alleviate the problem of sample imbalance.
  The experimental results of the above model on the test set are exposed in Table 6. Compared with the model Att-ON-LSTM without adding labeled semantic information, the accuracy of the model improves by 0.78% after adding labeled word directions. The macro-average F1 value is improved by 1.04%. Its shows that the semantic information of tag words is helpful to improve the effect of sentiment classification. However, the improvement effect is limited because the semantic information of the labeled words is not sufficient. After using the label semantic expansion process to enrich the semantic features of the labels, the accuracy of the model is improved by 1.79%. The average F1 value of the macro was improved by 2.02%. It indicates that the label semantic expansion process planned for this paper is effective. The optimized model ON-LSTM-LS using the cross-entropy loss function with weights has further improved the effect of emotion classification. Its accuracy is improved by 5.83% relative to the baseline model Att-ON-LSTM. The macro-average F1 value is improved by 6.38%, further demonstrating that the ON-LSTM-LS model can effectively improve the effectiveness of text sentiment analysis.
B. Comparative experimental results and analysis of similar models
  This group of experiments compares the ON-LSMT-LS model with related similar models to confirm the validity and accuracy of the ON-LSTM-LS model planned in this chapter. The comparison models are as follows:
  (1) LSTM: Long Short-Term Memory Network Model, which encodes text sequences from front to back. It is able to learn the distant information in the text and extract the one-way semantic features of the text.
  (2) CNN: Convolutional neural network model, which is able to extract n-elements features at different locations in a sentence by convolutional operation. It is able to learn textual relations within a certain distance through pooling operation and has an advantage over LSTM in terms of training speed.
  (3) LSTM-CNN: a hybrid model of long- and short-term memory network and convolutional neural network, the long- and short-term memory network is used to extract the global features of text. Convolutional neural network is used to extract local features of text, and then complete the learning of text contextual features.
  (4) Bi-LSTM: Bi-directional long- and short-term memory network model, which encodes text sequences from both positive and negative directions. It can learn the association relationship between the preceding and following words in the text and extract the contextual semantic features of the text.
  (5) Att-Bi-LSTM: Emotion analysis model based on Bi-LSTM and attention mechanism, which adds attention mechanism to the bidirectional long and short-term memory network. Its makes the model pay more attention to the words that have more influence on the classification effect during the learning process.
  (6) ON-LSTM: ordered neuron long and short-term memory network model. It is able to extract the text semantics while extracting the hierarchical syntactic information of the text to obtain a more comprehensive text feature.
  “The experimental results of the above models on the test set are exposed in Table 7. The model ON-LSTM-LS planned in this paper has a substantial improvement in accuracy and macro-average F1 value compared with similar baseline model LSTM, CNN, LSTM-CNN, Bi-LSTM, and Att-Bi-LSTM. The accuracy is improved by 19.79, 13.36, 11.95, 9.5, and 5.79%, respectively. Its shows that using three-layer ON-LSTM to extract semantic features of emotional text and combining them with semantic features of labels can improve the effectiveness of emotional classification more substantially. Compared with ON-LSTM and Att-ON-LSTM, the accuracy is improved by 9.04 and 5.28%, respectively. The macro-average F1 value score was improved by 6.87 and 6.38%. The study illustrates that the semantic information of labels is helpful for the improvement of sentiment analysis. As a whole, the ON-LSTM-LS model planned in this paper outperforms similar models for sentiment classification. The study shows that the ON-LSTM-LS model can effectively perform the text sentiment classification task with certain advantages.

**Table 6:** Results of ablation experiments.

**Models**	**Accuracy**	**Macro-F1**
ON-LSTM+Attention **(baseline)**	0.4691	03300
ON-LSTM+Attention+Label	0.4769	0.3404
ON-LSTM+Attention+Label+Label Extra	0.4870	03502
ON-LSTM+Attention+Label+Label Extra+Cutom Loss (**ours**)	**0.5229**	**0.3938**
ON-LSTM+Attention **(baseline)**	0.4691	03300

**Table 7:** Experimental results of comparing similar models.

**Models**	**Accuracy**	**Macro-F1**
LSTM	03,250	0.0764
CNN	0.3893	0.2747
LSTM-CNN	0.4034	0.2633
Bi-LSTM	0.4279	03582
Att-Bi-LSTM	0.4650	0.3264
ON-LSTM	0.4325	0.3251
Att-ON-LSTM	0.4691	03300
ON-LSTM-LS (**ours**)	**0.5229**	**0.3938**

**Figure 2:**
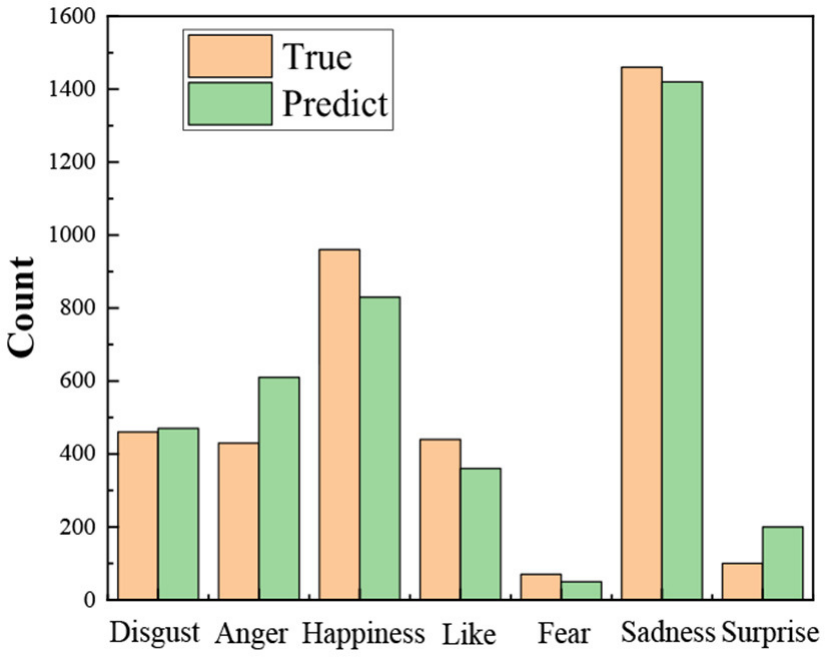
Distribution of forecasted categories and true categories.

Figure 2 shows the distribution of the predicted and true results of the ON-LSTM-LS model for different categories on the test set. The difference between the actual and predicted values of the seven emotions is small. However, due to data imbalance, the model has better classification results for two categories, disgust and sadness, than the prediction results for the remaining five categories. And the prediction effect of two emotions, happy and angry, was poor. In the next work, different methods should be explored to alleviate the data imbalance problem.

## Conclusion

How to extract this effective information from the huge amount of web texts and then apply it effectively in real life. This problem has led to the emergence of dynamic user text sentiment analysis techniques. To address the problem that syntactic information is ignored in emotional text feature extraction, this paper proposes a text feature extraction process based on ON-LSTM and attention mechanism. It is proved that it can better extract the semantic and hierarchical information in the emotional text and obtain more comprehensive emotional text features. The experimental results show that the sentiment analysis process based on ON-LSTM and tag semantics can effectively complete the text sentiment analysis task and improve the sentiment classification effect to a certain extent. The specific findings of the study are as follows:

(1) Att-ON-LSTM, compared with the Bi-LSTM model with added attention mechanism, improved the micro-average F1 value by 2.27% and the macro-average F value by 1.7%. The text features extracted by Att-ON-LSTM are more comprehensive and can effectively improve the effect of text emotion classification;
(2) The experimental results of ON-LSTM and the sentiment analysis model with labeled semantics on the test set showed a 0.78% improvement in the accuracy of the model with the addition of labeled word directions compared to the model Att-ON-LSTM without the addition of labeled semantic information. The macro-average F1 value was improved by 1.04%;
(3) The accuracy of the model improved by 1.79% after the label semantic features were enriched using the label semantic expansion process. The macro-average F1 value was improved by 2.02%. The sentiment classification of the model ON-LSTM-LS, which was optimized using the cross-entropy loss function with weights, was further improved. Its accuracy was improved by 5.83% relative to the baseline model Att-ON-LSTM.

## Data availability statement

The raw data supporting the conclusions of this article will be made available by the authors, without undue reservation.

## Author contributions

QZ planned the idea of revising the paper, designed the comparison experiment, and analyzed the results, then wrote the paper.

## Conflict of interest

The author declares that the research was conducted in the absence of any commercial or financial relationships that could be construed as a potential conflict of interest.

## Publisher's note

All claims expressed in this article are solely those of the authors and do not necessarily represent those of their affiliated organizations, or those of the publisher, the editors and the reviewers. Any product that may be evaluated in this article, or claim that may be made by its manufacturer, is not guaranteed or endorsed by the publisher.
